# Fishbone perforating Meckel’s diverticulum: an acute appendicitis mimicker

**DOI:** 10.1093/jscr/rjae293

**Published:** 2024-05-07

**Authors:** Sujan Kafle, Varsha Chhetri, Binesh Jha, Nabin Bhujel, Rambabu Khadka, Subhash Kumar Das

**Affiliations:** Provincial Hospital Bhadrapur, Nepal; B.P. Koirala Institute of Health Sciences, Nepal; Provincial Hospital Bhadrapur, Nepal; Provincial Hospital Bhadrapur, Nepal; Provincial Hospital Bhadrapur, Nepal; Provincial Hospital Bhadrapur, Nepal

**Keywords:** Meckel’s diverticulum, fishbone perforation, acute appendicitis

## Abstract

Perforation of Meckel’s diverticulum by a foreign body is rare, but when it occurs, it can mimic acute appendicitis, leading to diagnostic challenges. We present a case of a 45-year-old male initially diagnosed with acute appendicitis, but intra-operative exploration revealed a perforated Meckel’s diverticulum with a fish bone. Meckel’s diverticulum perforation remains diagnostically elusive, highlighting the need for intra-operative vigilance in cases of inconsistent findings like the presence of bilious fluid in the abdominal cavity. This case report underscores the importance of considering perforated Meckel’s diverticulum in the differential diagnosis of right iliac fossa pain and the necessity of surgical exploration for atypical presentations to ensure timely diagnosis and appropriate management.

## Introduction

Meckel’s diverticulum is a relatively common gastrointestinal abnormality caused by incomplete obliteration of the omphalomesenteric duct, which usually occurs within the first 9 weeks of gestation. It is a true diverticulum, containing all three layers of the bowel wall, and it originates at the antimesenteric border [1]. Although most patients with Meckel’s diverticulum are asymptomatic throughout their lives, ~4%–6% of the patients develop complications, including gastrointestinal bleeding, intestinal obstruction, intussusception, diverticulitis, enteroliths, perforation, fistula, and tumors [2].

Perforation of Meckel’s diverticulum by a foreign body is an extremely rare occurrence [3]. There have been a few cases described in the literature where perforation of Meckel’s diverticulum occurred due to a fish bone [4–8]. Perforated Meckel’s diverticulum can mimic acute appendicitis [8]. The Alvarado score, with a positive predictive value of ~90%, as described in the literature, is one of the commonly used scoring systems to diagnose acute appendicitis. It incorporates the patient’s history, clinical examination, and a few laboratory findings to aid in the presumptive diagnosis of acute appendicitis [9]. We present a case of a 45-year-old male patient who was diagnosed with acute appendicitis based on clinical, laboratory, and imaging findings, but running of the bowels due to inconsistent intra-operative findings of the appendix, led to the discovery of perforated Meckel’s diverticulum.

## Case report

A 45-year-old male presented to the Emergency Department of a community hospital in Eastern Nepal with a history of pain in the right lower quadrant for 1 day. He also complained of a fever not associated with chills and rigor, nausea, and anorexia. On examination, his vitals were: blood pressure—120/80 mmHg, pulse rate—105 beats per minute and regular, temperature—100.2°F, spO2—98% in room air, and respiratory rate—14/min. He had tenderness and rebound tenderness in the right iliac fossa. The labs showed leukocytosis. The Alvarado score was calculated to be 7/10.

The patient had undergone an ultrasound scan of his abdomen and pelvis at a private hospital outside, and it showed an inflamed appendix that was ~8 mm in diameter with no peri-appendiceal collection/lump. There was no evidence of a foreign body in the abdomen.

**Figure 1:**
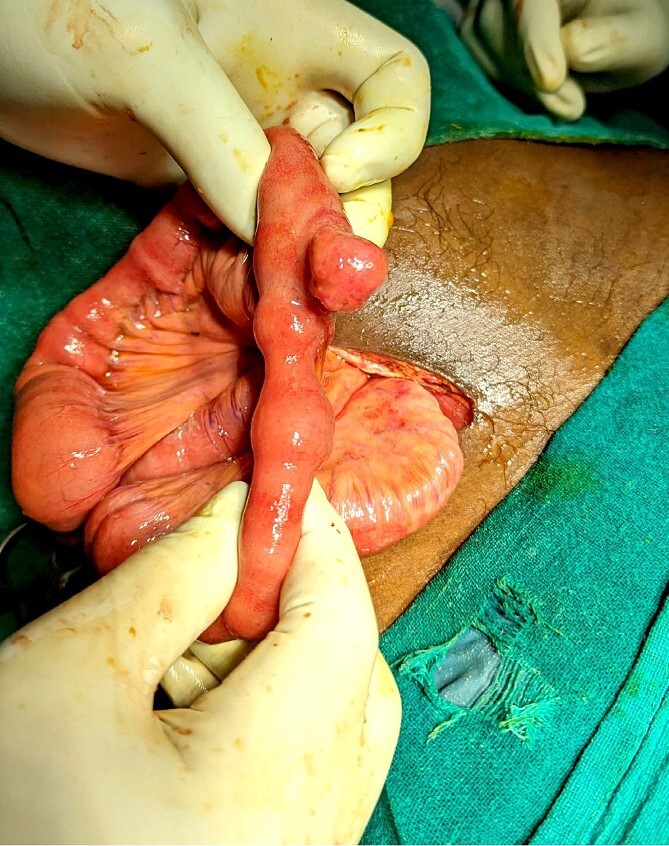
Intraoperative discovery of Meckel’s diverticulum during running of the bowels.

**Figure 2:**
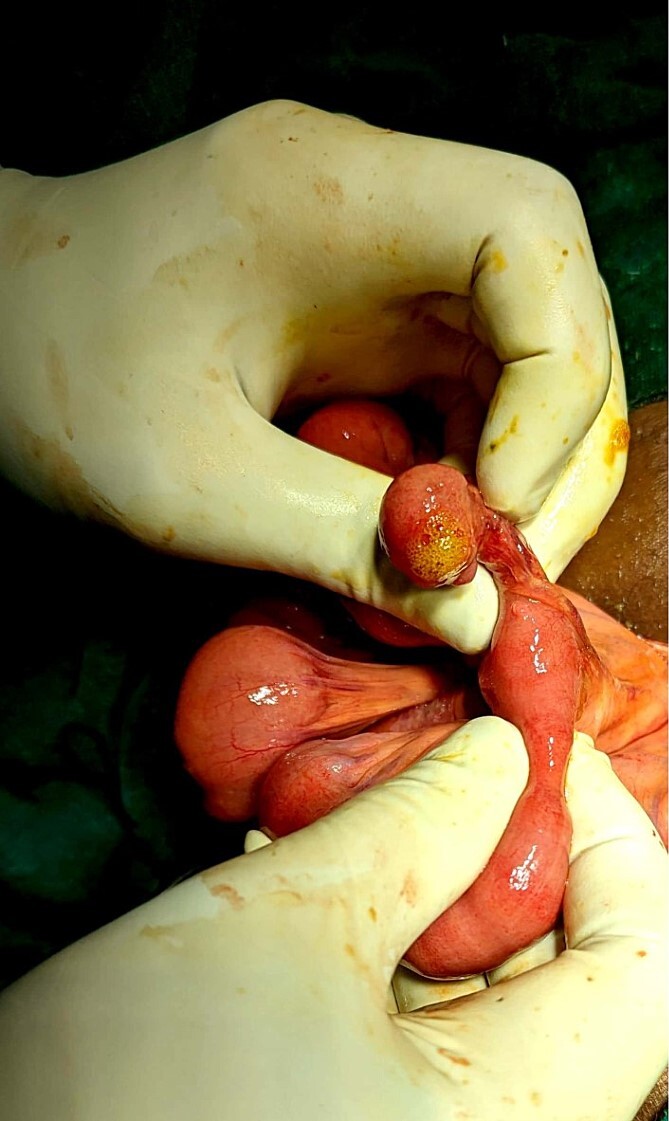
Bubbles coming out of the Meckel’s diverticulum during compression, suggesting perforation in the diverticulum.

**Figure 3:**
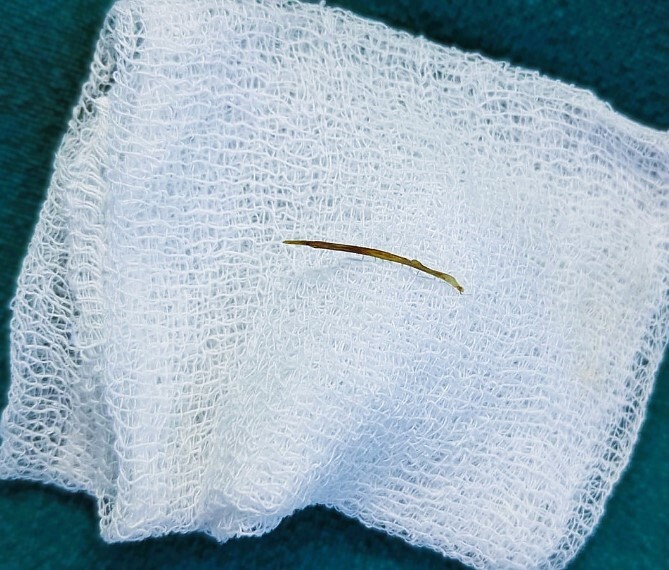
Fish bone revealed from the abdominal cavity.

He was managed in the Emergency Department with IV fluids, antibiotics, and NSAIDs, and it was planned for an emergency appendectomy after a few hours. He was taken to the operation theater, and a Gridiron incision was made. The appendix was mildly inflamed and had no perforation. But there was ~100 ml of bilious fluid in the abdominal cavity, and therefore, running of the bowels was performed to look for possible perforation elsewhere in the small intestine. The running of the bowels revealed Meckel’s diverticulum at ~50 cm from the ileocecal junction (Fig. 1). There was a perforation in the tip of the MD (Fig. 2). An appendectomy with Meckel’s diverticulectomy with anastomotic repair with peritoneal lavage and drain placement was performed. During the bowel wash, a sharp object was felt in the intraperitoneal cavity, and upon revealing it out of the abdomen, it was found to be a fish bone (Fig. 3).

On postoperative questioning, the patient gave a history of consuming fish 3 days back.

## Discussion

Meckel’s diverticulum is a true diverticulum that arises because of the failure of the obliteration of the vitellointestinal duct, which normally occurs within 9 weeks of gestation [1]. Less than 10% of Meckel’s diverticulum is diagnosed before surgery, and it is mostly found incidentally during the surgery [10].

Meckel’s diverticulum is very difficult to diagnose both clinically and radiologically, and imaging features are non-specific [8].

Some cases of Meckel’s diverticulum can be complicated, and Meckel’s diverticulitis accounts for 13%–31% of such cases [11]. Perforation of Meckel’s diverticulum is very rare and accounts for 0.5% of symptomatic cases. When perforation occurs, it mainly occurs due to foreign bodies, inflammation, or trauma. There are multiple foreign bodies reported as a cause of perforation of Meckel’s diverticulum, and fish bones are one of the important causes of perforation, accounting for around 55% of the cases [1].

A condition called Valentino’s syndrome has been described in the literature, where perforation of the peptic ulcer leads to leakage of the digestive fluid, which seeps and settles down in the right iliac fossa, causing inflammation and leading to signs and symptoms of acute appendicitis [12]. Although Valentino’s syndrome has not been described in relation to the perforation of Meckel’s diverticulum, it can lead to leakage of the digestive fluid, which might cause appendicular inflammation in a similar fashion.

Although rare, perforation of Meckel’s diverticulum can present as right iliac fossa pain, mimicking acute appendicitis [8]. This case report is a reminder to the treating physicians that perforation of Meckel’s diverticulum should be kept as a differential diagnosis of acute appendicitis, even when the scoring systems like Alvarado score, and ultrasonography of the abdomen may suggest acute appendicitis. Solely relying on these tools can lead to missing out on the search and repair of the perforation, which can result in fatal peritonitis later on. Also, this case report highlights the importance of exploring the bowel during appendectomy, for possible perforation, when bilious fluid is present in the abdominal cavity in the setting of a non-perforated appendix.

## Conclusion

The complications of Meckel’s diverticulum are rare, and foreign body perforation is one of the rarest complications among those. However, when it does occur, it can mimic acute appendicitis, and even scoring systems and imaging may fail to distinguish it from acute appendicitis. Therefore, it should be considered as one of the differential diagnoses of right iliac fossa pain and should be explored intra-operatively if there are inconsistent intraoperative findings.

## Supplementary Material

km_20240326_1080p_30f_20240326_223339_rjae293
